# REAL-WORLD USE OF BOTULINUM TOXIN-A FOR POST-STROKE SPASTICITY IN THE NETHERLANDS: A RETROSPECTIVE CLAIMS STUDY

**DOI:** 10.2340/jrm.v58.43952

**Published:** 2026-01-15

**Authors:** Max VAN WIJK, Mary VERHOEVEN, Thom S. LYSEN, Hanne VAN BALLEGOOIJEN, Alexander C.H. GEURTS

**Affiliations:** 1 IQVIA, Amsterdam; 2 Ipsen, Hoofddorp; 3 Department of Rehabilitation, Radboud University Medical Center, Nijmegen, the Netherlands

**Keywords:** botulin toxin A, post stroke spasticity, real-world evidence, epidemiology, reimbursement claims

## Abstract

**Objective:**

The aim of this observational study was to describe the real-world use of botulinum toxin-A in patients with a stroke in the Netherlands.

**Methods:**

This study used longitudinal insurance claims data between 2012 and 2016 with 30% nationwide coverage and included patients with both stroke-related and botulinum toxin-A claims. All analyses were descriptive and displayed as summary statistics.

**Results:**

60,222 patients with a stroke were identified, of whom 18,141 (30.1%) were treated in a rehabilitation centre or hospital and 1.7% (1,036 patients) were treated with botulinum toxin-A. A total of 2,855 botulinum toxin-A claims from 890 patients were included in the analysis (1.5% of all patients in the database). Mean age was 63.4 (SD ± 14.0) years at index injection cycle, with a median follow-up of 3.7 years (interquartile range [IQR] 2.2–4.5 years). Patients received up to 16 injection cycles with a median of 2 injection cycles (IQR 1–5 cycles). The median Time-To-Next-Injection-Cycle was 17 weeks (IQR 13–25 weeks). The total number of injection cycles was 604 for abobotulinumtoxinA (22.1%) and 2,251 (78.8%) for onabotulinumtoxinA. Doses per cycle ranged from 200–660 for abobotulinumtoxinA and 101–400 units for onabotulinumtoxinA.

**Discussion:**

Claims data from a national healthcare insurance fund in the Netherlands showed that only 1.7% of patients with post-stroke spasticity received botulinum toxin-A. Botulinum toxin-A treatment doses and intervals varied widely across patients and most patients received only 1 or 2 botulinum toxin-A injection cycles. Our results suggest undertreatment of spasticity with botulinum toxin-A and suboptimal treatment adherence in Dutch clinical practice.

## LAY ABSTRACT

After a stroke, patients can experience muscle stiffness, which is known as post-stroke spasticity. Botulinum toxin-A injections are commonly used to reduce symptoms, but real-world treatment patterns are not well studied. This study examined how botulinum toxin-A is used in patients with post-stroke spasticity in the Netherlands using insurance claims from 2012–2016. Of 60,222 patients with a stroke, only 1.7% were treated with botulinum botulinum toxin-A. A total of 2,855 botulinum toxin-A claims from 890 patients (1.5% of all patients in the database) were included in this study. These patients had an average age of 63.4 years at the time of the first botulinum toxin-A injection. Patients received up to 16 treatment cycles, with an average of 2 cycles and an interval of 17 weeks between cycles. Two formulations of botulinum toxin-A were available in the Netherlands between 2012 and 2016. Most patients (76.9%) received onabotulinum toxin-A at an average dose of 290 units, while the remaining patients received abobotulinum toxin-A at an average dose of 500 units. It is important to note that the dosing recommendations for onabotulinum toxin-A and abobotulinum toxin-A differ and cannot be directly compared. This study suggests potential undertreatment of spasticity with botulinum toxin-A and suboptimal treatment adherence in the Netherlands.

Stroke is a leading cause of disability globally and incidence rates are increasing (1). The incidence and prevalence of stroke in the Netherlands are estimated to be about 40,000 and 370,000, respectively (2). Patients can develop spasticity after stroke, which is an abnormal, involuntary muscle activity caused by damage to motor neurons in the central nervous system (3, 4). Spasticity can affect both upper and lower limbs and is often painful and disabling. Approximately 25% to 40% of stroke patients develop spasticity at any time point post-stroke (3, 5, 6), while the prevalence of disabling spasticity post stroke is estimated to range from 2% to 13% (7).

The management of spasticity is challenging due to differences in patient profiles and treatment expectations. The standard of care usually consists of a combination of physical and pharmacological interventions, often using a variety of different approaches according to individual patients’ needs (8–10). Botulinum toxin-A (BoNT-A) is an effective treatment for reducing spasticity in adults post-stroke, improving motor symptoms and pain (5, 11, 12). Spasticity guidelines recommend the use of BoNT-A for focal spasticity that has resulted in passive or active movement impairment leading to activity limitations. Clinical guidelines advise to treat patients with BoNT-A through a coordinated multidisciplinary approach, involving physical management and therapy (8, 9, 11). Treatment effects may vary per individual, but typically last for 3 to 4 months and, therefore, patients require repeated injection cycles (13–17).

Although evidence on the efficacy and safety of BoNT-A has been well established in the setting of clinical trials (17–19), there is limited knowledge on how patients with post-stroke spasticity are treated in the long term with BoNT-A in real-world clinical practice. In observational analyses from other countries, only predefined patient groups were studied, e.g., patients with spasticity 6 months after stroke or with spasticity in specific muscle groups (13, 20, 21). Few real-world studies have reported on the treatment of BoNT-A in post-stroke patients (22–29). Gaps persist in our understanding with regard to the real-world treatment patterns and long-term utilization of BoNT-A among a broader population of patients with post-stroke spasticity and in the Netherlands. There is, thus, a need for broader stroke population-based studies that report on real-world use of BoNT-A treatment for spasticity.

The aim of this study was to describe the real-world use of BoNT-A in patients with post-stroke spasticity using insurance claims data in the Netherlands. A Dutch healthcare insurance claims database was used with 30% nationwide coverage (approximately 6 million people) between 2012 and 2016.

## METHODOLOGY

### Study design and inclusion/exclusion criteria

This retrospective, cohort study used longitudinal insurance claims data to describe real-world BoNT-A use in patients with post-stroke spasticity in the Netherlands. The study included patients with any stroke-related healthcare claim (codes listed in Table SI) during the period from 1 January 2012 to 31 December 2016, aged 18 years or older at first BoNT-A injection cycle. Patients received at least 1 injection of BoNT-A (abobotulinumtoxinA [aboBoNT-A; Dysport^®^] or onabotulinumtoxinA [onaBoNT-A; Botox^®^]; codes listed in Table SII) with a diagnosis code for stroke in the rehabilitation setting (Fig. S1).

### Data sources

Claims were derived from IQVIA’s Hospital Claims Database (HCD). The HCD holds pseudonymized claims for healthcare activities from medical specialist care in the Netherlands, collected for financial purposes. Claims follow the Dutch diagnosis-related group classification scheme (“DBC”), and contain diagnostic codes, demographic information, and codes for expensive (“add-on”) medication such as BoNT-A.

Claims covered by the HCD involve all sites where medical specialist inpatient and outpatient care is provided, including hospitals and rehabilitation centres. Claim coverage was approximately 30% nationwide, with limited regional differences. Proportional coverage varied across healthcare sites. The HCD includes a population considered nationally representative in terms of age and sex.

### Baseline characteristics

Baseline characteristics included patient demographics (age, sex), hospital name and location, information on diagnosis codes, injection cycle, and medication. The index date was defined as the date of the first injection cycle with BoNT-A.

### Follow-up

Patients were followed up from first occurrence of any claim in the HCD (not restricted to stroke, rehabilitation, or BoNT-A), until end of the follow-up period (31 December 2016). The insurance claims data did not cover loss to follow-up, treatment discontinuation, safety data, or death.

### Outcomes

Outcomes from this study included (*i*) descriptive information on number of patients with stroke, age at the occurrence of stroke, and/or index injection of BoNT-A, (*ii*) the mean/median time between stroke and the first post-stroke BoNT-A treatment for spasticity, and the (*iii*) mean/median dose and number of injection cycles of BoNT-A per brand as treatment for post-stroke spasticity. Drug-related covariates were listed as brand, administration form, and dose (i.e., units of BoNT-A). The costs per injection cycle per patient were calculated based on the pharmacy purchase price in the Netherlands as of April 2023 (i.e., €1.80 per unit onaBoNT-A and €0.36 per unit aboBoNT-A) for the complete study period.

### Statistical methods

In this observational study, no formal statistical testing was performed, and all analyses were descriptive by nature. Summary statistics included number of observations, number of missing data, mean and standard deviation (SD), median and first and third quartiles, minimum and maximum, and 95% confidence intervals (CI) for means. Proportions were based on the number of non-missing observations. Repeated measures were aggregated by first aggregating claims on patient level by taking the sum of events per patient divided by patient-years of follow-up (per-patient-per-year [PPPY]) and provided as group-level summary. Where appropriate, analyses were stratified by BoNT-A brand. Injection cycles were ranked across patients (1st injection cycle for all patients, 2^nd^ injection cycle, up to the nth injection cycles with corresponding dates per patients) to further describe dose and the Time-To-Next-Injection-Cycle (TTNIC). Descriptive statistical analyses were performed using R software version 4.1.1 (R Foundation for Statistical Computing, Vienna, Austria).

This study was performed in accordance with the Declaration of Helsinki and in compliance with national laws and Good Pharmacoepidemiology Practices (GPP). Ethical approval was obtained from the Dutch institutional review board (reference number NWMO22.07.017). The authors of this study followed the STROBE guidelines for reporting of observational studies.

## RESULTS

### Study population

**Fig. 1:**
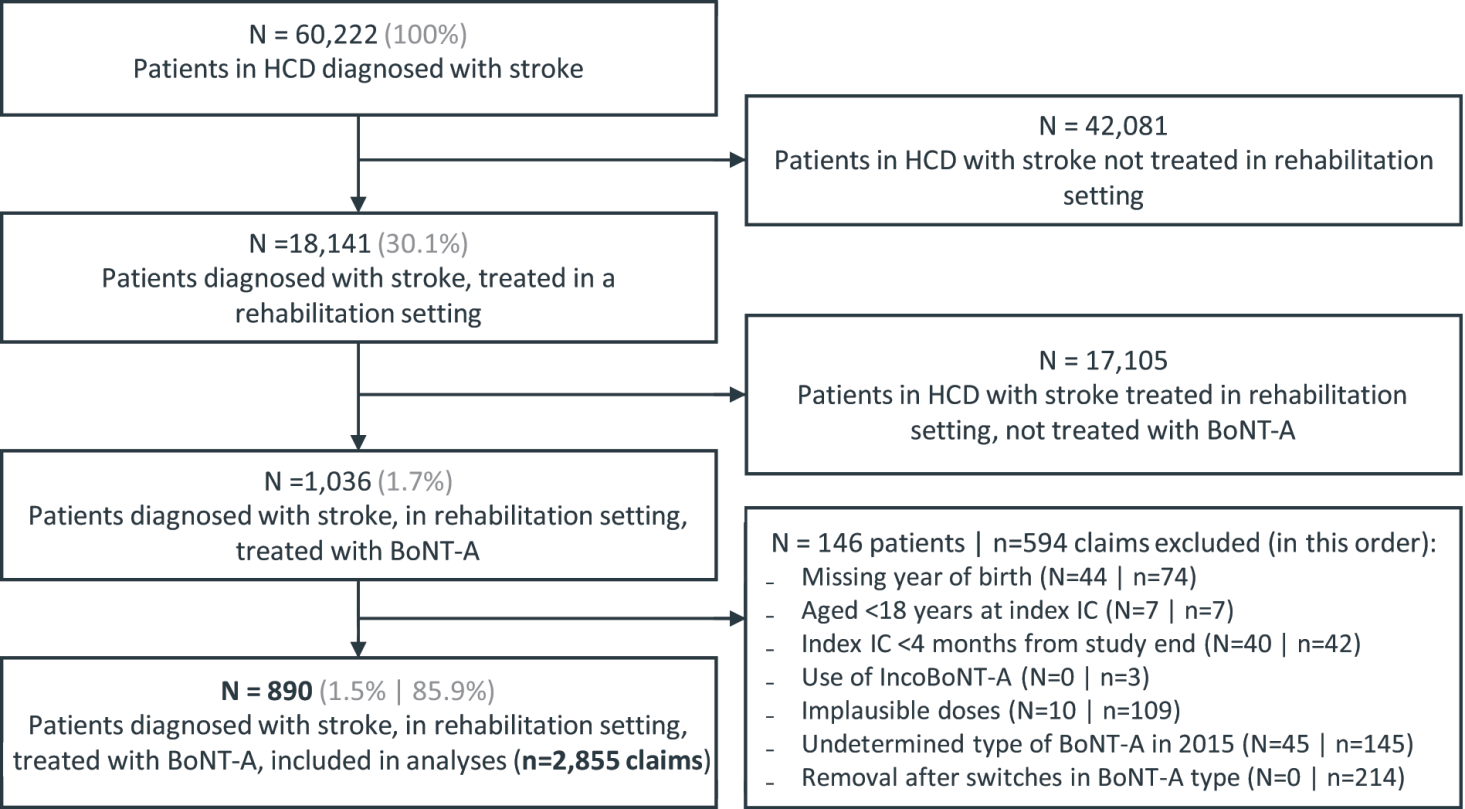
Inclusion of study population. Selection of the study population took place at the claims level, i.e., a diagnosis of stroke was determined by that patient having any stroke-related healthcare claims throughout the study period. In selecting the final group of patients for analyses, exclusion of claims did not necessarily result in excluding that patient. To illustrate, an individual patient was not excluded if they switched to incoBoNT-A or another BoNT-A brand. BoNT-A: botulinum toxin-A. HCD: hospital claims database, IC: injection cycle; *N* = number of patients, *n* = number of claims.

This study identified 60,222 patients with stroke-related healthcare claims in the HCD (enrolled population). One-third (30.1%; 18,141 patients) were treated in a rehabilitation centre or a rehabilitation department in a hospital, and 1.7% of all patients (1,036 patients) were treated with BoNT-A. A total of 2,855 claims by 890 patients (1.5%) were included in the analyses (Fig. 1). The mean age of the enrolled population (*n* = 60,222) was 71.2 (SD ± 14.6) years at the first stroke-related claim. Patients in the analytical sample (*n* = 890) were younger with a mean age of 63.4 (SD ± 14.0) years at index injection cycle. Half (49.9%) of all patients were female (Table I). Patients were followed up for a median (interquartile range [IQR]) of 3.7 years (2.2–4.5 years) from the first BoNT-A claim to last claim for any treatment during the study period. Claims from patients were distributed largely evenly over the study period, regardless of the brand of BoNT-A (Table SIII).

**Table I:** Characteristics of study population

Characteristic	Enrolled population (*n* = 60,222)	Study population (*n* = 890)
Age at first stroke-related claim (enrolled population) or at index injection cycle (study population), years, mean ± SD	71.2 ± s14.6	63.4±14.0
Median (IQR), range	73.5 (62.5–82.1)	64.7 (54.5–73.6)
Range		19.7–96.1
Female (%)	29,135 (48.4%)	444 (49.9%)
Follow-up time from first claim to last claim, in patient-years		2,914
Cumulative follow-up time for all patients, median (IQR) per patient	NA	3.7 (2.2–4.5)
Range per patient		0.0–5.0

IQR: interquartile range; NA: not applicable.

### Treatment characteristics

The majority of patients were treated with onaBoNT-A (*n* = 684; 78.8%) and the remainder were treated with aboBoNT-A (*n* = 206; 21.2%). The overall, median (IQR) time between the first stroke claim and the index injection cycle was 3.8 months (0–14.4) for all patients (*n* = 890).

### Injection characteristics

Across follow-up, 2,855 injection cycles over 2,914 patient-years were observed, with a median (IQR) of 1.0 injection cycle per-patient-per-year (PPPY; 0.5–2.1; Table II), considering inter-individual varying follow-up. Over the entire study period, patients received a median (IQR) of 2.0 injection cycles (1–5).

**Table II:** Descriptive results of BoNT-A injection cycles across follow-up

Characteristic	Study population (*n* = 890)
*n*	2,855
N/patient-year	1.0
*n* per patient, median (IQR)	2 (1–5)
*n* per patient, range	1–23
PPPY, median (IQR)	1.0 (0.5–2.1)
Number of injection cycle per patient, median (IQR)	2 (1–5)
BoNT-A brand: aboBoNT-A, *n* (%)	604 (21.2%)
BoNT-A brand: onaBoNT-A, *n* (%)	2,251 (78.8%)

IQR: interquartile range; aboBoNT-A: abobotulinumtoxinA (Dysport^®^); onaBoNT-A: onabotulinumtoxinA (Botox^®^); PPPY: per-patient-per-year.

**Fig. 2:**
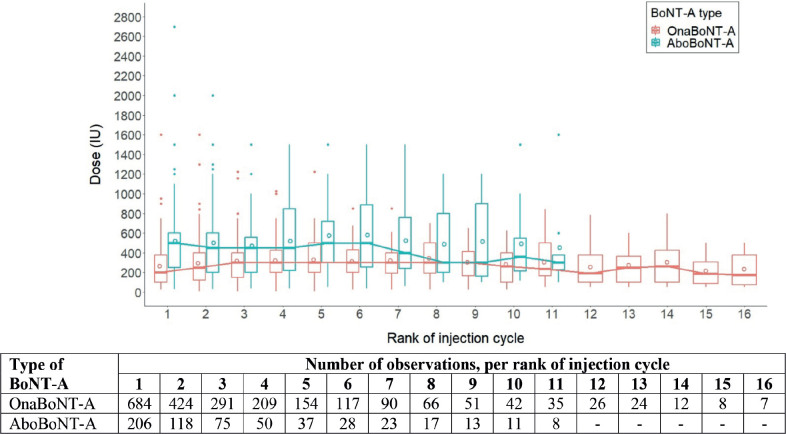
Number of injection cycle, per brand of BoNT-A.

Injection cycles were ranked over time per patient to further describe injection patterns. More than one-third of all patients received only 1 injection cycle (*n* = 348; 39.1%). A second injection cycle was given to 542 patients were given to 542 patients (60.9% of total patients), and 366 patients (41.1% of total patients) received ≥ 3 injection cycles (Fig. 2).

The TTNIC was determined among 542 patients who had ≥ 2 injection cycles. The median (IQR) TTNIC combined for both brands was 17 weeks (13–25 weeks), with a wide range of 1–172 weeks per injection cycle (Table III). For aboBoNT-A, the median (IQR) TTNIC was 15 weeks (13–21); for onaBoNT-A the median was 18 weeks (14–26 weeks).

**Table III:** Descriptive results of time to next injection cycle across follow-up

Characteristic	Brand of BoNT-A	Measure/unit	Values (*n* = 542 patients, *n* = 1,965 intervals between injection cycles)
Time to next injection cycle (weeks), per injection cycle	Both	*n* (% of total IC)	1,965 (68.8%)
		Median (IQR)	17.0 (13.4–25.1)
		Range	1.1–171.7
	AboBoNT-A	*n* (% of total intervals)	398 (20.3%)
		Median (IQR)	15.0 (13.0–21.5)
		Range	3.0–171.7
	OnaBoNT-A	*n* (% of total intervals)	1,567 (79.7%)
		Median (IQR)	17.9 (14.0–26.0)
		Range	1.1–166.0
Time to next injection cycle (weeks), median per patient	Both	*n* (% of study population)	542 (60.1%)
		Median (IQR)	19.2 (14.0–31.9)
		Range	1.1–171.7
		*n* (% of patients with ≥ 3 ICs)	366 (41%)
	AboBoNT-A	*n* (% of patients with ≥ 2 ICs)	118 (21.7%)
		Median (IQR)	16.9 (13.0–25.8)
		Range	3.0–171.7
	OnaBoNT-A	*n* (% of patients with ≥ 2 ICs)	424 (78.2%)
		Median (IQR)	20.1 (14.0–32.6)
		Range	1.1–166.0

aboBoNT-A: abobotulinumtoxinA (Dysport^®^); onaBoNT-A: onabotulinumtoxinA (Botox^®^); IQR: interquartile range.

**Fig. 3:**
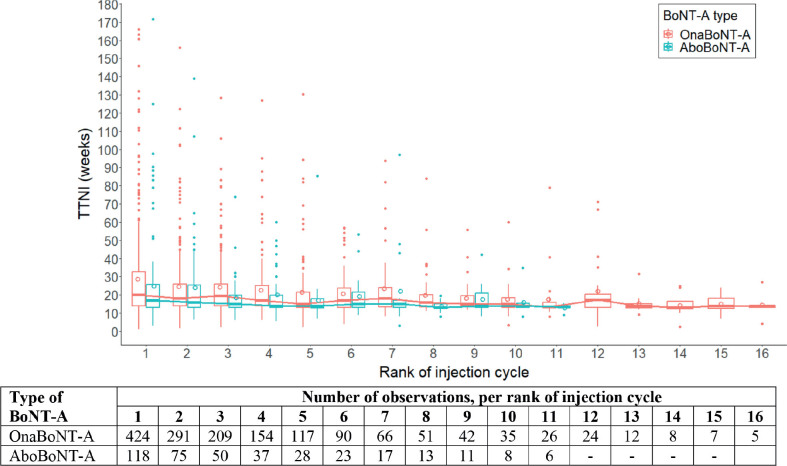
Boxplots of time to next injection cycle across ranked injection cycles, per brand of BoNT-A. Time to next injection cycle in weeks is shown across ranked injections, where the first rank concerned the interval between injection cycle no. 1 and injection cycle no. 2. Boxplots show the variability per rank, per brand (median, hinges [25^th^ and 75^th^ percentile], and whiskers [5^th^ and 95^th^ percentile]). Means are also plotted (open circles), per rank. Closed dots are outliers beyond the 5th and 95th percentiles, per rank.

The TTNIC was highly variable per rank (i.e., number of injection cycle per patient). The greatest outliers were present in the earlier ranked injection cycles although large intervals were still noticeable for patients with ≥ 10 injection cycles (Fig. 3).

### Dose characteristics

Stratified per brand of BoNT-A, the total number of injection cycles was 604 for aboBoNT-A (22.1%), and 2,251 for onaBoNT-A (78.8%). The median (IQR) dose for aboBoNT-A per injection cycle was 500 U (200–660). For onaBoNT-A, the median dose was 290 U (101–400). When adjusting for intra-patient variability, the median dose per patient was similar to the overall median dose per injection cycle for both aboBoNT-A at 500 U (300–600) and onaBoNT-A at 229 U (150–400) (Table IV).

**Table IV:** Descriptive results of BoNT-A dose of injection cycles across follow-up

Brand of BoNT-A	Characteristic	Measure	Values (*n* = 890 patients, *n* = 2,855 injection cycles)
AboBoNT-A	Dose per injection cycle	Median (IQR)	500 (200–660)
		Range	30–2,700
		% above advised clinical max. dose 1,500 U	4 (0.7%)
		Number of observations	604
OnaBoNT-A	Dose per injection cycle	Median (IQR)	290 (101–400)
		Range	10–1,600
		% above advised clinical max. dose 600 U	185 (8.2%)
		Number of observations	2,251
AboBoNT-A	Dose per injection cycle – median per patient	Median (IQR)	500 (300–600)
		Range	32–2,700
		% above advised clinical max. dose 1500 U	2 (1.0%)
		Number of observations	206
OnaBoNT-A	Dose per injection cycle – median per patient	Median (IQR)	229 (150–400)
		Range	10–1,600
		% above advised clinical max. dose 600 U	56 (8.2%)
		Number of observations	684

Doses of BoNT-A were summarized across all *n* = 2,855 injection cycles, or across all *n* = 890 patients for the “per patient’ measures. Doses were provided in International Units. Note, this study considered doses outside of the following ranges as outliers, which were excluded before analyses: 25 to 5,000 for aboBoNT-A, and 10 to 5,000 for onaBoNT-A (inclusive dose ranges).

aboBoNT-A: abobotulinumtoxinA (Dysport^®^); IC: injection cycle; onaBoNT-A: onabotulinumtoxinA (Botox^®^); IQR: interquartile range; SD: standard deviation; U: international units.

**Fig. 4:**
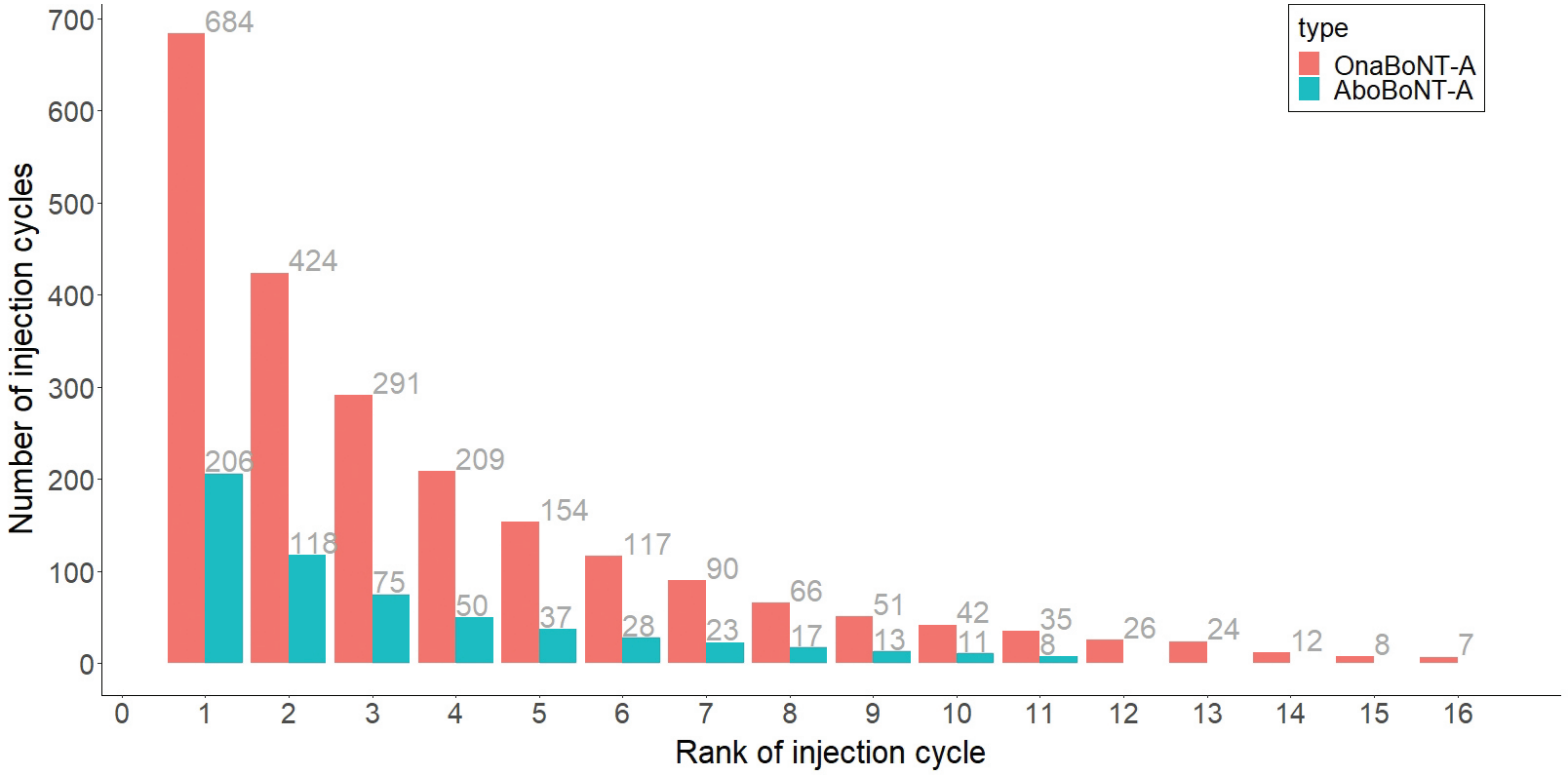
Boxplots of dose of BoNT-A across ranked injection cycles, per brand of BoNT-A. Dose is shown across ranked injections, where the first rank concerned injection cycle no.1. Boxplots show variability per rank, per brand (median, hinges [25^th^ and 75^th^ percentile], and whiskers [5^th^ and 95^th^ percentile]). Means are also plotted (open circles), per rank. Closed dots are outliers beyond the 5th and 95th percentiles, per rank. The boxplots include only rank in which sufficient observations are present; we excluded *n* = 5 ranks for onaBoNT-A and *n* = 6 for aboBoNT-A.

Dose across ranked injection cycles varied widely per brand of BoNT-A. For aboBoNT-A, doses per patient remained relatively stable after initiation of few consecutive ranks and showed a decrease from rank 7 onwards. For onaBoNT-A, there was a slight increase in doses across the first 3 ranked injection cycles and plateaued afterwards (Fig. 4). Variability in TTNIC per rank across patients was lower for patients who had ≥ 4 injection cycles, especially for aboBoNT-A (Fig. S3).

### Treatment costs

The overall median (IQR) cost (cost-year 2023) for BoNT-A per patient was €631 (€180–722) in total for all injection cycles in the analysed period. The median cost per patient per injection cycle was €361 (€180–541) across follow-up (Table SIV). The overall median cost per injection cycle for aboBoNT-A was €182 (€109–219) and EUR 413 (271–721) for onaBoNT-A.

## DISCUSSION

In this retrospective study using claims data from the Netherlands between 2012 and 2016, we described the real-world use of BoNT-A in patients treated for post-stroke spasticity in a rehabilitation centre or a rehabilitation department in a hospital. A total of 60,222 patients with a stroke were identified, of whom 18,141 (30.1%) were treated in a rehabilitation centre or hospital and 1.7% (1,036 patients) were treated with BoNT-A. During the study period, 60% of all treated patients received 1 or 2 injection cycles. Doses and TTNIC varied considerably between individuals and across time.

The small proportion (1.7%) of patients diagnosed with stroke and treated in a rehabilitation setting who were treated with BoNT-A suggests underuse of botulinum toxin-A in clinical practice as a treatment for post-stroke spasticity. Previous studies reported a prevalence of post-stroke spasticity of 25–40% (3, 5, 6), while severe or disabling spasticity occurs in approximately 2–13% of all post-stroke patients (7). These findings suggest that the proportion of patients suffering from post-stroke spasticity, who are eligible for treatment with BoNT-A, is substantially higher than the proportion of patients (1.7%) who were treated with BoNT-A in our study. Findings from a nationwide study on rehabilitation care in the Netherlands in 2022 reported that the majority of patients with stroke were discharged from the acute hospital setting to their home situation for follow-up. Only a small proportion of patients with stroke received care in the subacute phase coordinated by a medical specialist in a rehabilitation facility, usually with outpatient follow-up after discharge (30). An analysis of outpatient claims data from Germany (*n* = 7,947) reported a similar proportion of patients with post-stroke spasticity (1.0%) who were treated with BoNT-A (27). A lack of awareness regarding post-stroke spasticity among physiotherapists and general practitioners, which has previously been reported by 2 survey-based studies among general practitioners in Germany (28, 29), may explain the high proportion of patients who were not treated in a rehabilitation setting. The prevalence of patients with stroke and disabling spasticity in the community is relatively low, and spasticity-related problems may not often be recognized by general practitioners. The current guidelines on stroke for physiotherapists and general practitioners in the Netherlands provide only limited guidance on spasticity management after stroke (31, 32). Physiotherapists, occupational therapists, general practitioners, and specialists in elderly care may therefore benefit from appropriate screening tools to identify and refer patients for proper management of their spasticity after stroke. Although the current study shows that a large proportion of patients with stroke were seen by a rehabilitation specialist (30.1%), it remains unclear why the proportion of patients treated with BoNT-A was so low.

In the current study, the mean age of patients with stroke was 71.2 years while patients treated with BoNT-A were younger with a mean age of 63.4 years. The mean age of patients with stroke in our study is in line with similar studies from Germany (71–74 years) (22, 27, 29). As in our study, results from other observational studies also indicate that patients treated with BoNT-A are generally younger than the overall patient population with post-stroke spasticity (23–25).

So far, limited real-world studies have reported on the treatment of BoNT-A in patients with post-stroke spasticity and even fewer have reported on costs. To our knowledge, this is the first study to report on real-world treatment patterns of BoNT-A among patients with stroke in the Netherlands.

Comparable results regarding treatment patterns were found in a small retrospective chart review study (*n* = 189) in the United States among patients admitted between 2014 and 2018 for inpatient rehabilitation within a 6 months post-stroke (25). Our results are also in line with a recent observational study from France (*n* = 138,481) covering nationwide hospital discharge records. Although the latter sample was a more heterogeneous selection of patients with central nervous system lesions, about half (53.5%) of all patients received 1 or 2 BoNT-A injections (26). In another retrospective analysis (*n* = 2,452) using healthcare claims from 2015–2019 in the United States, it was also observed that adult patients received 2.5 injection cycles on average (24). The relatively low mean or median number of repeated injection cycles across different studies is remarkable, given that BoNT-A treatment is meant to be carried out over a longer period of time to be optimally effective. The fact that treatment with BoNT-A is prematurely terminated in most patients may indicate that careful assessments of treatment effect take place in clinical practice, after which the treatment with BoNT-A is stopped after only 1 or 2 ineffective treatment cycles. On the other hand, it may indicate a lack of adherence to BoNT-A treatment, despite established efficacy. The observed lack of adherence and the underusage of BoNT-A may have multiple causes that can either be patient (e.g., insufficient motivation, frailty, pain, fear) or healthcare (e.g., lack of physician follow-up, limited treatment resources) related. General practitioners in Germany previously reported the lack of available physicians who can administer BoNT-A as the main reason for not treating patients with BoNT-A, followed by poor medical indications, patient refusal, and inadequate reimbursement from healthcare insurance companies (28, 29).

An analysis based on the international ASPIRE study described real-world treatment patterns (spanning two years) of 411 post-stroke spasticity patients using onaBoNT-A. About one-third (29.9%) of patients were categorized as treatment nonadherent, defined as patients who received less than 2 injection cycles (23). Being treated in a European centre and the use of orthotics were associated with adherence, defined as patients who were treated with ≥ 3 treatment cycles with onaBoNT-A during 2-year follow-up. Characteristics associated with nonadherence were a TTNIC of ≥ 15 weeks and moderate-to-severe disability on an upper-limb disability pain scale. The mean treatment interval was 18.1 weeks for adherent patients and 23.6 weeks for nonadherent patients, indicating large variations in treatment patterns (23). The ULIS-III study was a prospective, multi-country (14 countries) cohort study following adult patients over 2 years regarding upper-limb spasticity management. The majority had post-stroke spasticity (91% of 1,004 patients) and the most frequent therapy intervention after BoNT-A treatment was passive stretch (70–80%). The median duration of the BoNT-A treatment was 4 cycles. AboBoNT-A showed a significant longer treatment duration compared with onaBoNT-A (13). The study provided evidence for a sustained functional benefit of repeated cycles of BoNT-A as measured by both person-centred goal setting and improvements in standardized measures.

In our retrospective study, the TTNICs were on average longer than in randomized controlled trials (15–17), but shorter than in the prospective ULIS-III study that featured extensive goal setting and found a mean TTNIC of 25 weeks (13). The lack of capacity to administer BoNT-A injections in certain treatment centres may be one of the reasons to explain why intervals were longer than in clinical trials. The variability in TTNIC may also be explained by the need for goal-oriented and personalized treatment plans and patient preferences.

The financial burden of stroke in the Netherlands was previously estimated at €29,484 per patient (cost-year 2012) in the first year after a stroke, of which less than 2% (€506) was attributable to medication (33). In the Netherlands, medicines are considered expensive if the (anticipated) expenditure per patient exceeds the threshold of €1,000 per year (34). Although BoNT-A is considered as an “expensive medicine” in the Netherlands, BoNT-A does not meet this criterion based on the results of this study, irrespective of the brand used. The costs calculated in this study are in line with more recent data from 2018 to 2023, which show that the average expenditure on BoNT-A per patient per year in the Netherlands was between €440 and €490 (35). It must be noted that this is the average expenditure on all patients who are treated with BoNT-A and these figures are specific to the treatment of post-stroke spasticity.

As mentioned earlier, the relatively low level of treatment adherence observed in the current study may be explained by a variety of patient- and healthcare-related causes, which warrants further real-world research in this area. Another still unmentioned reason for non-adherence is the fact that BoNT-A – at least in the Netherlands – is increasingly used as a short-term treatment as bridging therapy to long-term surgical interventions, such as selective neurectomies and soft-tissue orthopaedic surgery (e.g., tendon lengthening or transfer). The use of individualized measurement tools such as the Goal Attainment Scale (GAS) or the Canadian Occupational Performance Measurement (COPM) tool to improve communication, patient understanding, and expectation of the treatment was not so common at the time of the current study but may also influence treatment adherence. A qualitative research study on patients with chronic spasticity after stroke found that patients prefer shared decision-making in spasticity management, particularly regarding the timing of BoNT-A injections, which may improve treatment adherence (36). A recent study demonstrated that such online monitoring may be feasible for implementation in Dutch clinical practice (37).

This is the first study that describes the real-world use of BoNT-A in post-stroke spasticity in the Netherlands. Our findings are congruent with the personalized application of BoNT-A in routine healthcare of patients with post-stroke spasticity. The data represent a large proportion of the Dutch population in the hospital claims database. It must, however, be noted that this study describes a patient population based on claims from 2012 to 2016. The management of spasticity in the Netherlands may have changed after publication of the national guideline on treatment of cerebral and/or spinal spasticity in 2016 (8).

Several methodological considerations should be mentioned. Due to the retrospective study design, it was not possible to determine the reason for BoNT-A treatment termination. In addition, the number of injection cycles per patient may be underestimated as it is likely that some patients were lost to follow-up, or this may be due to missing data as patients might have switched to another healthcare insurance company. The annual percentage of healthcare insurance switchers in the Netherlands is, however, relatively low (6–9% per year) (38). The claims database covers all sites where medical specialist in- and outpatient rehabilitation care is provided, including hospitals and rehabilitation centres, but it was not possible to distinguish between in- and outpatient administrations. Lastly, we chose to define follow-up time based on available data of any healthcare claim in the hospital claims database. There is, thus, the possibility that the true follow-up time per patient may be under- or overestimated.

As known from published literature and prescribing information, BoNT-A brands cannot directly be compared in terms of dose because dose recommendations are specific to the brand of BoNT-A (ona-/aboBoNT-A). The BoNT-A dosage per muscle should be based on patient weight, the size of the muscle, the number of affected muscles, effect of the last BoNT-A treatment, spasticity severity, and pre-set treatment goals (5, 11). Due to the nature of these hospital claims data, it was not possible to evaluate the specific muscles targeted in the BoNT-A treatment or spasticity severity. It was also not possible to determine whether goal setting was used or whether other types of (supportive) therapy were applied. These factors can all influence the dosage used, TTNIC, and treatment effectiveness.

In conclusion, this is the first study to describe real-world treatment use of BoNT-A in patients who were treated for post-stroke spasticity in the Netherlands between 2012 and 2016. Only 1.7% of patients with post-stroke spasticity received BoNT-A, suggesting undertreatment in clinical practice. BoNT-A treatment doses and intervals varied widely across patients, which suggests that BoNT-A was used as a personalized treatment. The fact that most patients received only 1 or 2 BoNT-A injection cycles may indicate that treatment adherence is not optimal in Dutch clinical practice.

## Supplementary Material




