# Supplementary material for: REAL-WORLD USE OF BOTULINUM TOXIN-A FOR POST-STROKE SPASTICITY IN THE NETHERLANDS: A RETROSPECTIVE CLAIMS STUDY
Source: J Rehabil Med. 2026 Jan 15;58:43952. doi: 10.2340/jrm.v58.43952 (PMC12814239; doi:10.2340/jrm.v58.43952)
Supplement: Supplementary file 1 [file JRM-58-43952-s1.pdf]

Supplementary material has been published as submitted. It has not been copyedited, or typeset by Journal of Rehabilitation Medicine

Table SI. Diagnostic codes used to identify claims with stroke-related healthcare

**Medical specialty**		**Diagnostic code**	
**Stem**		**Appendix**	**Label**
0330	Neurologist	1111	Ischemic stroke
		1102	Intracerebral haemorrhage
0308	Neurosurgeon	1230	Intracerebral hematoma, supratentorial, non-traumatic, evacuation by craniotomy
		1235	Intracerebral hematoma, infratentorial, non-traumatic: evacuation by craniotomy
		1240	Decompression cerebral infarction by means of craniotomy and possibly dural dilatation
0327	Rehabilitation	0313	CVA
0335	Geriatrist	0263	CVA/TIA
8418	Specialist elderly care	101	CVA

Abbreviations: CVA=Cerebrovascular Accident; TIA=Transient Ischemic Attack.

Table SII. Healthcare product codes (‘overig zorgproduct’ [OZP]) used to extract claims of BoNT-A from the claims database

OZP code	OZP description
193317	Botulinum toxin (dysport), injection powder, per used unit of 1U used for the indication which is registered on the substance name in the NZa performance and tariff table for add-on medicines.
193316	Botulinum toxin (botox), injection powder, per used unit of 1U used for the indication which is registered on the substance name in the NZa performance and tariff table for add-on medicines.
193358	Botulinum toxin, injection powder, per unit of 1U used for the indication which comply with the policy rule for performances and rates of specialty care.
193483	Botulinum toxin, injection powder, per unit of 50U used for the indication which comply with the policy rule for performances and rates of specialty care.
193484	Botulinum toxin, injection powder, per unit of 100U used for the indication which comply with the policy rule for performances and rates of specialty care.
193486	Botulinum toxin, injection powder, per unit of 300U used for the indication which comply with the policy rule for performances and rates of specialty care.
193487	Botulinum toxin, injection powder, per unit of 500U used for the indication which comply with the policy rule for performances and rates of specialty care.
193314	Botulinum toxin (azzalure), injection powder, per used unit of 1U used for the indication which is registered on the substance name in the NZa performance and tariff table for add-on medicines.

Table SIII. Number of claims and number of patients throughout study period, overall and stratified per calendar year

**Characteristic**	**Overall study period**	**Stratified per calendar year**					
		**2012**	**2013**	**2014**	**2015**	**2016***	**2016****
Number of BoNT-A claims per period (N)	2,855	565	521	619	576	574	615
OnaBoNT-A claims (N; % of total claims)	2,251 (78.8%)	450 (79.6%)	410 (78.7%)	500 (80.8%)	448 (77.8%)	443 (77.2%)	470 (76.4%)
AboBoNT-A claims (N; % of total claims)	604 (21.2%)	115 (20.4%)	111 (21.3%)	119 (19.2%)	128 (22.2%)	131 (22.8%)	145 (23.6%)
Number of patients with any BoNT-A claims (N)	890	320	311	299	308	296	335
With OnaBoNT-A claims (N; % of total patients)	684 (76.9%)	246 (76.9%)	244 (78.5%)	227 (75.9%)	245 (79.5%)	235 (79.4%)	260 (77.6%)
With AboBoNT-A claims (N; % of total patients)	206 (23.1%)	74 (23.1%)	67 (21.5%)	72 (24.1%)	63 (20.5%)	61 (20.6%)	75 (22.4%)
Number of patients with their first claim per time period (N)	-	320	180	175	133	82	121
With OnaBoNT-A claims (N; % of total patients)	-	246 (76.9%)	134 (74.4%)	139 (79.4%)	100 (75.2%)	65 (79.3%)	90 (74.4%)
With AboBoNT-A claims (N; % of total patients)	-	74 (23.1%)	41 (22.8%)	41 (23.4%)	33 (24.8%)	17 (20.7%)	31 (25.6%)
Number of patients with their last claim per time period (N)	-	163	143	165	123	296	335
With OnaBoNT-A claims (N; % of total patients)	-	118 (74.2%)	106 (74.1%)	125 (75.8%)	100 (81.3%)	235 (79.4%)	260 (77.6%)
With AboBoNT-A claims (N; % of total patients)	-	45 (27.6%)	37 (25.9%)	40 (24.2%)	23 (18.7%)	61 (20.6%)	75 (22.4%)

*Numbers of injection cycles in 2015 may be lower since brand of BoNT-A was not provided and this could only be determined for a subgroup of claims that year; excluding 145 undetermined BoNT-A injection cycles resulted in excluding 45 patients from analyses. *Patients with the first BoNT-A claims in the last 4 months of 2016 were excluded. **Additional analyses with all claims from patients who were treated with BoNT-A in 2016.*

Abbreviations: aboBoNT-A=abobotulinumtoxinA (Dysport®); onaBoNT-A=onabotulinumtoxinA (Botox®); N=number of patients.

Table SIV. Cost of BoNT-A for post-stroke spasticity, stratified across brand and calendar years

**Characteristic**	**Overall study population (N=890)**	**OnaBoNT-A (n ICs=2,251; n patients=684)**	**AboBoNT-A (n ICs=604; n patients=206)**
Costs–overall follow-up			
Costs per injection cycle			
Median (Q1-Q3)	631 (180-722) €	523 (182-722) €	182 (73-240) €
Range	11-2886 €	18-2886 €	11-983 €
Median cost per injection cycles per patient			
Median (Q1-Q3)	361 (180-541) €	413 (271-722) €	182 (109-219) €
Range	12-2886 €	18-2886 €	12-983 €
Costs per injection cycles - Stratified per calendar year			
2012			
Median (Q1-Q3)	361 (180-559) €	442 (180-722) €	146 (77-182) €
Range	11-1431 €	18-1431 €	11-546 €
2013			
Median (Q1-Q3)	361 (180-722) €	541 (180-722) €	182 (73-182) €
Range	12-1624 €	18-1624 €	12-546 €
2014			
Median (Q1-Q3)	361 (180-722) €	510 (182-722) €	164 (73-228) €
Range	11-1443 €	43-1443 €	11-546 €
2015			
Median (Q1-Q3)	361 (180-631) €	451 (194-722) €	155 (73-328) €
Range	15-2345 €	72-2345 €	15-983 €
2016			
Median (Q1-Q3)	364 (182-722) €	541 (325-767) €	182 (109-328) €
Range	15-2886 €	45-2886 €	15-728 €

Costs were calculated multiplying cost per unit, taking cost prices from the pharmacy purchase price of April 2023 (€1.80 per unit onaBoNT-A and €0.36 per unit aboBoNT-A). Separate calculations per BoNT-A brand were summed to provide overall costs, and provided separately. Cost per calendar year included claims of BoNT-A for that year; costs were not aggregated per patient first and injection cycles from one patient could contribute to multiple years.

Abbreviations: aboBoNT-A=abobotulinumtoxinA (Dysport®); U=units; onaBoNT-A=onabotulinumtoxinA (Botox®); Q=quartile; SD=standard deviation; ICs=injection cycles.
