# Supplementary material for: REAL-WORLD USE OF BOTULINUM TOXIN-A FOR POST-STROKE SPASTICITY IN THE NETHERLANDS: A RETROSPECTIVE CLAIMS STUDY
Source: J Rehabil Med. 2026 Jan 15;58:43952. doi: 10.2340/jrm.v58.43952 (PMC12814239; doi:10.2340/jrm.v58.43952)
Supplement: Supplementary file 2 [file JRM-58-43952-s2.pdf]

Supplementary material has been published as submitted. It has not been copyedited, or typeset by Journal of Rehabilitation Medicine

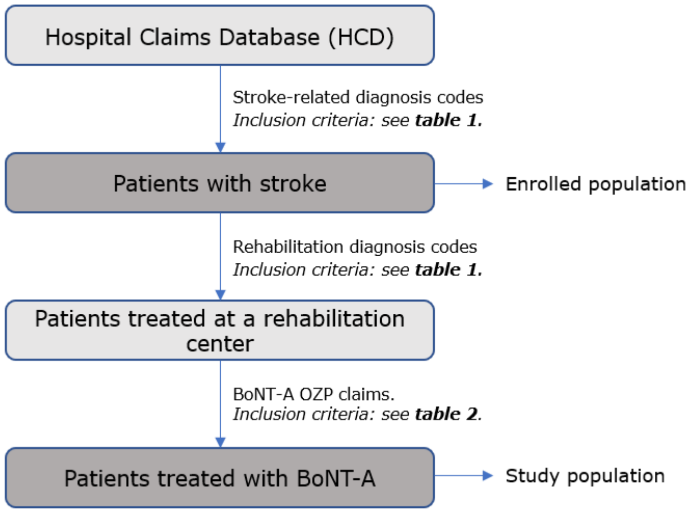

Fig. S1. Flowchart of planned selection of claims, and study population, from the Hospital Claims Database

Planned selection of the relevant claims and corresponding study population from the HCD. After selecting on stroke-related healthcare claims, claims indicative of stroke treatment in the rehabilitation setting, and BoNT-A, claims and corresponding patients were eligible for selection into the study population (further data selection and cleaning steps to be taken).

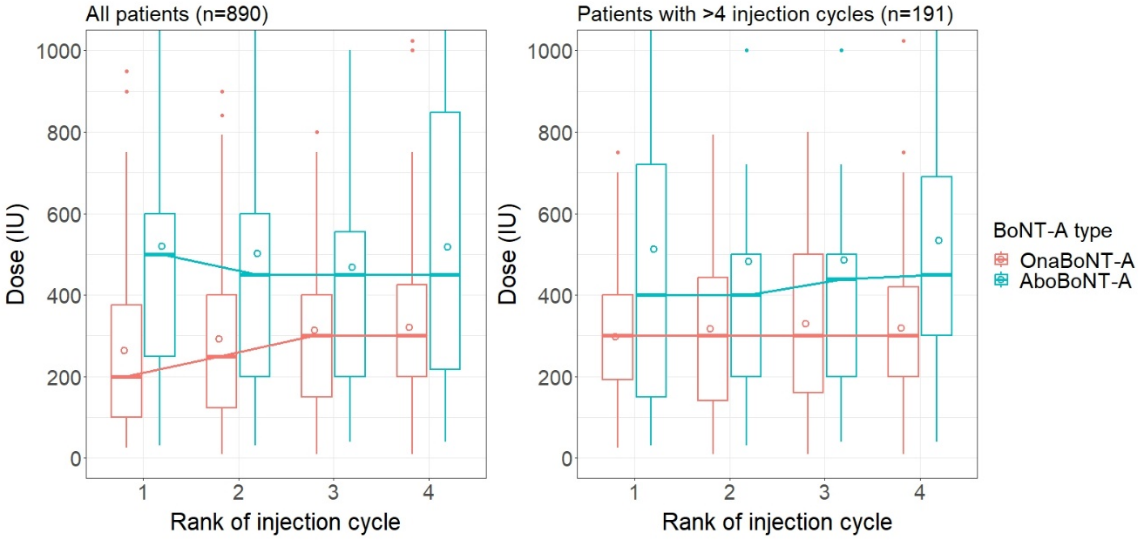

Fig. S2: Boxplots of dose across ranked injection cycles, per brand of BoNT-A, limited to first 4 injection cycles for all patients and patients with >4 cycles

Doses per brand of BoNT-A are shown for the first 4 injection cycles, for all patients (left panel) and for the subgroup selected on having more than 4 injection cycles throughout follow-up (right panel). Differences per brand between the 2 panels show to what extent time to next injection cycle was driven by patients who received only a few injection cycles. The split at ≤4 versus >4 injection cycles was chosen arbitrarily. Numbers of observations for the right panel across ranked injection cycles 1 through 4 are all the same per rank, per brand: n=154 for onaBoNT-A, and n=37 for aboBoNT-A. Note, the Y-axis is limited to 1050 U.

Abbreviations: BoNT-A=botulinum toxin-A; U=International Units.

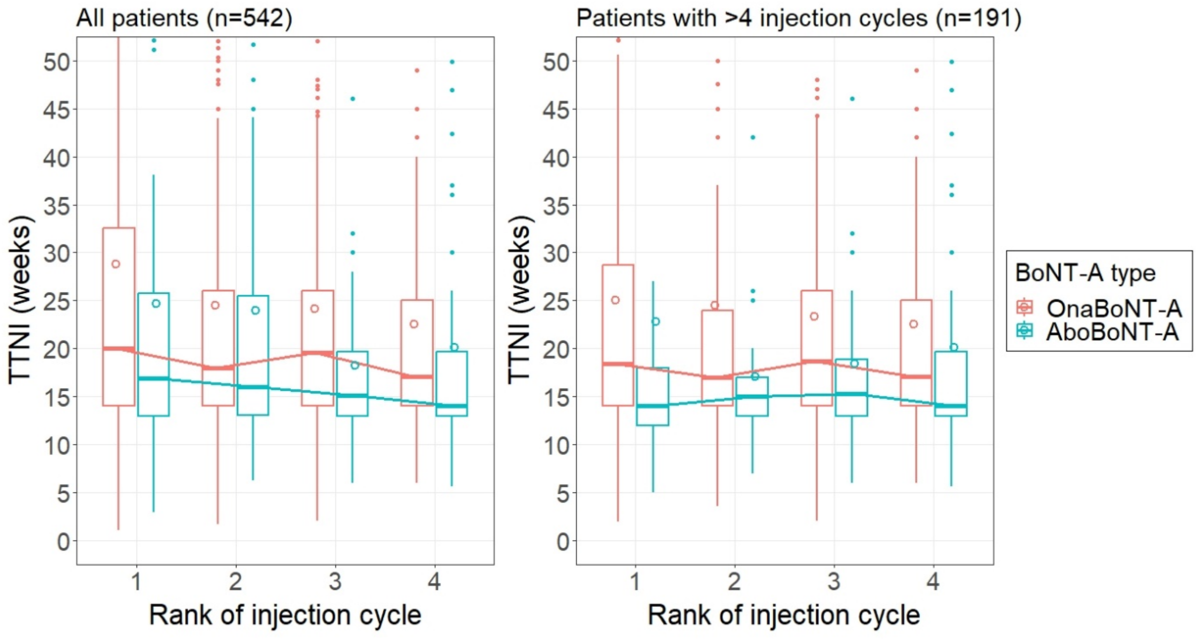

Fig. S3: Boxplots of TTNIC across ranked injection cycles, per brand of BoNT-A, limited to first 4 injection cycles for all patients and patients with >4 cycles

The time to next injection cycle is shown for the first 4 injection cycles, for all patients (left panel) and for the subgroup selected on having more than 4 injection cycles throughout follow-up (right panel). Differences per brand between the 2 panels show to what extent time to next injection cycle was driven by patients who received only a few injection cycles. The split at ≤4 versus >4 injection cycles was chosen arbitrarily. Numbers of observations for the right panel across ranked injection cycles 1 through 4 are all the same per rank, per brand: n=154 for anaBoNT-A, and n=37 for aboBoNT-A. Note, the Y-axis is limited to 55 weeks
